# The International, Prospective COSMOS (CytOSorb® TreatMent Of Critically Ill PatientS) Registry: Interim Results in Patients with Septic Shock

**DOI:** 10.1016/j.aicoj.2026.100052

**Published:** 2026-03-19

**Authors:** Ricard Ferrer, Thomas Kirschning, Moritz Unglaube, Ulf Guenther, Julian Kreutz, Matthias Thielmann, Andreas Baumann, Andreas Kribben, Dietrich Henzler, Nuno Germano, Aschraf El-Essawi, Filippo Aucella, Thomas Guenther, Martin Bellgardt, Bartosz Tyczynski, P. Christian Schulze, Gabriella Bottari, Jorge Hidalgo, Jean-Louis Teboul, Dana Tomescu, Teresa Klaus, Weihong Fan, Joerg Scheier, Efthymios N. Deliargyris, Fabio Silvio Taccone

**Affiliations:** a Intensive Care Department, Vall d'Hebron University Hospital, Shock, Organ Dysfunction and Resuscitation Research Group (SODIR), Barcelona, Spain; b Department of Cardiothoracic Surgery, Heart and Diabetes Center NRW, Bad Oeynhausen, Germany; c Department of Intensive Care, Helios Dr. Horst- Schmidt Klinik Wiesbaden, Wiesbaden, Germany; d University Hospital of Anesthesiology and Intensive Care, Klinikum Oldenburg, Oldenburg, Germany; e Department of Cardiology, Angiology, and Intensive Care Medicine, University Hospital, Philipps University of Marburg, Marburg, Germany; f Department of Thoracic and Cardiovascular Surgery, West German Heart & Vascular Center Essen, University Hospital Duisburg-Essen, Essen, Germany; g Department of Anaesthesiology, Intensive Care Medicine and Pain Management, BG University Hospital Bergmannsheil, Medical Faculty of Ruhr University Bochum, Bochum, Germany; h Department of Nephrology, University Duisburg-Essen, University-Hospital Essen, Essen, Germany; i Department of Anesthesiology, Surgical Intensive Care, Emergency and Pain Medicine, Ruhr-University Bochum, Klinikum Herford, Herford, Germany; j Department of Intensive Care, Hospital Curry Cabral, Lisboa, Portugal; k Department of Thoracic and Cardiovascular Surgery, University Medical Center Goettingen, Goettingen, Germany; l Department of Nephrology and Dialysis, "Casa Sollievo della Sofferenza" Foundation, Scientific Institute for Research and Health Care, San Giovanni Rotondo, Italy; m Department of Cardiovascular Surgery, German Heart Center Munich, School of Medicine & Health, TUM University Hospital, Technical University of Munich, Munich, Germany; n Department of Anesthesiology and Intensive Care Medicine, St Josef-Hospital Bochum, University Hospital of the Ruhr-University Bochum, Bochum, Germany; o Department of Medical Intensive Care, University Hospital Essen, Essen, Germany; p Department of Internal Medicine and Cardiology, University Hospital Jena, Jena, Germany; q Pediatric Intensive Care Unit, Children Hospital Bambino Gesù, IRCCS, Rome, Italy; r General Intensive Care Unit and COVID-19 Unit, Belize Healthcare Partners, Belize City, Belize; s Paris-Saclay Medical School, Paris-Saclay University, Le Kremlin-Bicêtre, France; t Department of Anesthesiology and Critical Care III, “Carol Davila" University of Medicine and Pharmacy, Fundeni Clinical Institute, Bucharest, Romania; u Medical Affairs, CytoSorbents Europe GmbH, Berlin, Germany; v Medical Affairs, CytoSorbents Corporation and CytoSorbents Medical Inc., Princeton, New Jersey, United States of America; w Department of Intensive Care, Hôpital Universitaire de Bruxelles (HUB), Université Libre de Bruxelles (ULB), Brussels, Belgium

**Keywords:** CytoSorb, Hemoadsorption, Hemoperfusion, Adsorption, Blood purification, Hyperinflammation, Sepsis, Septic shock, Registry

## Abstract

**Background:**

The international prospective COSMOS Registry (NCT05146336) collects real-world data on CytoSorb® (CS) hemoadsorption utilization patterns and outcomes in critically ill patients. This analysis focuses on patients with septic shock.

**Methods:**

Following informed consent, data was systematically collected before, during, and after CS treatment. Time frame of data collection was from the initiation of COSMOS study enrollment (July 15, 2022) to date of data extraction (May 7, 2025). Study follow-up extended to 90 days. We compared details on vasopressor requirements, fluid balance, and P/F ratio before and after CS treatment. APACHE II was assessed at ICU admission, while SOFA scores were determined at the start and end of CS therapy. Safety of the device was assessed based on investigator-reported device-related adverse effects. Data are presented as either mean ± standard deviation or as median with interquartile ranges (IQR).

**Results:**

A total of 140 patients (mean age of 61 ± 15 years, 33% female) at 18 study sites treated for septic shock was analyzed. On admission, median APACHE II score was 24 [18,30], SOFA score was 13 [11,15] and Charlson scores of 4 [2,6]. CS therapy was applied as part of kidney replacement therapy (KRT, 85%), standalone hemoperfusion (10%) or extracorporeal membrane oxygenation (ECMO, 5%). On average, each patient received 2.8 ± 2.2 adsorbers, with 47% receiving three or more. CS therapy was associated with a significant reduction in interleukin (IL)-6 levels (from 2,013 [219, 39,988] to 108 [75, 1,662] pg/mL, p < 0.0001) and noradrenaline requirement (0.23 [0.09, 0.43] to 0.07 [0.02, 0.22] µg/kg/min, p < 0.0001), reduced fluid balance (+1,386 [−15, 2,960] to +59 [−738, 1,614] mL, p < 0.0001), and improved oxygenation (P/F ratio 120 [70, 208] to 172 [114, 257], p = 0.0003). CS therapy was also associated with a significantly reduced platelet count (123 [86, 182] to 66 [37, 121] ×10^9^/L, p < 0.0001). Overall SOFA score did not change significantly (p = 0.65), however, system-specific SOFA scores improved significantly for respiratory, cardiovascular and renal sub-scores, while coagulation worsened. Observed ICU mortality was 32.4%. No serious adverse device effects or dysfunctions were reported.

**Conclusions:**

In this Registry, CytoSorb® therapy was associated with significant early clinical benefits in patients with septic shock, including hemodynamic stabilization and improved fluid balance. Further systematic research is needed to optimize its use and identify patient populations that benefit most.

## Background

Septic shock, representing the most severe form of sepsis, remains a leading critical and life-threatening condition worldwide, with a high prevalence in intensive care units (ICUs). Key features of septic shock include profound vasoplegia, refractory hypotension, and multi-organ dysfunction resulting from an overwhelming inflammatory response [1]. Early identification and appropriate management – fluid resuscitation, vasopressor support, and source control – are crucial components of therapy. Despite advances in supportive care and antimicrobial therapies, septic shock continues to carry significant mortality rates, ranging from 30 to 50% depending on the patient population and healthcare setting [2], highlighting the need for additional therapeutic approaches. CytoSorb® (CS) hemoadsorption therapy has emerged as an adjunctive intervention in the management of septic shock and systemic immune dysregulation [[3], [4], [5]]. CS is an extracorporeal blood purification device that utilizes a highly porous polymer adsorber to remove cytokines and other inflammatory mediators like pathogen- and damage-associated molecular patterns (PAMPs and DAMPs), from the bloodstream, thereby mitigating the excessive immune activation that contributes to organ failure [6]. The treatment aims to achieve hemodynamic stabilization by reducing vasopressor requirements thereby preserving organ function and improving overall clinical outcomes in critically ill patients.

The role of CS in septic shock is supported by growing evidence from both interventional studies and real-world clinical practice. Previous studies have demonstrated significant reductions in inflammatory markers [7] and positive effects on hemodynamics [8] when CS therapy was used in combination with standard care. However, randomized controlled trials (RCTs) are still pending (RCT PROCYSS NCT04963920), and further data are needed to establish best practices, optimal timing, and patient selection criteria for its use in septic shock. It is noted that evidence from septic shock RCTs have in general yielded disappointing results over the years, due in part to the complexity and heterogeneity of this condition [[9], [10], [11]].

Real-world evidence provides critical insights into the effectiveness and safety of therapies in broader, unselected patient populations. Registries play an essential role in capturing these data, offering a valuable complement to clinical trials by reflecting real-life practice and outcomes [12]. The COSMOS Registry, an international multicenter observational study, aims to evaluate the use of CS therapy in critically ill patients [13]. By collecting comprehensive real-world data, the Registry seeks to expand our understanding of the clinical benefits, treatment protocols, and outcomes associated with CS therapy. This interim analysis was conducted for patients with septic shock, a frequent indication in the Registry, to assess data quality, exclude safety concerns, and provide the first planned subgroup evaluation.

## Methods

### Registry study design

The COSMOS Registry (NCT05146336) is a prospective, international, multicenter observational study conducted in countries where the device has received approval for routine clinical application. Participating sites are chosen based on their established experience with the device, patient recruitment capacity, and commitment to the study. Device utilization is expected to adhere to established best practices [[14], [15], [16]], and all participating site staff receive thorough training to ensure standardized and appropriate use. Investigators are advised to consult the most recent Instructions for Use for details on contraindications and precautions. To minimize selection bias, sites are instructed to enroll all eligible patients consecutively without prior selection. Data collection begins only after obtaining approval from Institutional Review Boards (IRBs) or Independent Ethics Committees (IECs), and informed consent is secured from all participating patients. Among additional eligible critical care indications like rhabdomyolysis or liver failure, septic shock represents the largest cohort of the Registry so far.

Inclusion criteria:

1 CytoSorb® 300 mL device utilization
2 Informed consent

Exclusion criteria:

3 Use of the CytoSorb® 300 mL device for antithrombotic removal only
4 Intraoperative use of CytoSorb® 300 mL device during cardiac surgery only
5 The occurrence of a complication or other medically justified circumstance that arises after written informed consent has been obtained from the patient and before or during the planned therapy and as a result of which the use of CS adsorber is contraindicated or no longer appropriate.

This analysis includes 140 patients with septic shock [1] enrolled by 18 participating institutions from Germany, Italy, Spain, and Portugal who were treated with CS as part of routine clinical care.

### Study outcomes and data collection

Data were systematically collected at the following time points:

• 24 h before CS initiation
• During CS treatment
• 24 h post-treatment
• At ICU and hospital discharge
• At 90-day follow-up

The following section provides an overview of all baseline characteristics, treatment-related variables, laboratory and clinical parameters, as well as outcome and safety endpoints included in the analysis.

1 Demographics
• Age
• Sex
2 Comorbid Disease / Risk Scores
• APACHE II score (Acute Physiology and Chronic Health Evaluation) at ICU admission
• SOFA score (Sequential Organ Failure Assessment) at the start and end of CS therapy
• Charlson Comorbidity Index
3 Treatment Characteristics
• Type of extracorporeal circuit used
• Need for kidney replacement therapy (KRT)
• Number of CytoSorb® (CS) adsorbers used per patient
• Duration of CS treatment
• Timing of CS therapy initiation relative to Intensive Care Unit (ICU) admission
4 Laboratory Biomarkers (Pre- and Post-CS)
• Interleukin-6 (IL-6)
• C-Reactive Protein (CRP)
• Procalcitonin
• Lactate
• Creatinine
• pH
• Platelet count
• Albumin
• Bilirubin
5 Clinical Parameters (Pre- and Post-CS)
• Norepinephrine dosage
• Fluid balance
• Oxygenation (P/F ratio, ratio of partial pressure of oxygen in arterial blood to the fraction of inspiratory oxygen concentration)
• Need for invasive mechanical ventilation
• Mean arterial pressure (MAP)
• MAP/Norepinephrine index
6 SOFA Subscores (Organ-Specific, Pre- and Post-CS)
• Respiratory (P/F ratio)
• Cardiovascular (MAP or vasopressor use)
• Renal (Creatinine)
• Coagulation (Platelets)
• Liver (Bilirubin)
• Central Nervous System (Glasgow Coma Scale, GCS)
7 Mortality and other Outcomes
• ICU mortality
• ICU length of stay (in survivors)
• Need for KRT at 90-day follow-up
8 Safety Parameters
• Serious adverse device effects (SADE)
• Device deficiencies (DD)
• Bleeding events
• Need for albumin substitution

ICU mortality was assessed as the primary outcome. Furthermore, association of early versus late start of CS regarding onset of shock with mortality was investigated. Septic shock itself was defined according to the Sepsis-3 criteria, requiring vasopressor support to maintain MAP ≥ 65 mmHg together with a lactate level ≥2 mmol/L despite adequate fluid resuscitation [1], with onset of shock being defined as time point of admission to ICU. Also, patients with need for kidney replacement therapy (KRT) were analyzed separately from those without need of KRT. Device safety was assessed by investigator-reported adverse events related to the device until ICU discharge or death, whichever occurred first.

### Data quality

#### Data completeness

Data completeness is continuously monitored through a built-in report within the Electronic Data Capture (EDC) system, allowing real-time tracking of missing or incomplete data. In addition, data completeness is a standing agenda item during regular calls with study sites, where any outstanding documentation is identified and sites are reminded to complete data entry in a timely manner. However, due to the nature of the Registry, certain data remain unavailable, as only parameters collected during routine practice are recorded, and no additional data collection (e.g., laboratory values) is required by the protocol.

#### Monitoring procedures

This Registry Study employs a risk-based monitoring (RBM) approach that integrates multiple methods to ensure data quality and patient safety. The RBM strategy includes:

  - Statistical Monitoring: Detection of data outliers and anomalies through statistical analysis methods.
  - Centralized Data Review: Continuous, real-time evaluation of site data to identify implausible, inconsistent, or medically irrelevant entries. A dedicated medical review is performed by a physician with extensive experience in intensive care. Queries are raised with sites in case of inconsistencies.
  - On-Site Monitoring: Conducted by the Sponsor’s representative or designee to perform source data verification and assess study conduct at the site.

#### Data consistency

Automated edit checks are implemented within the EDC system to identify discrepancies and ensure internal consistency across all collected variables. When a discrepancy or implausible value is entered, the system automatically generates a query for resolution.

### Statistical analysis

Continuous variables are reported as median with interquartile range (IQR), or mean with standard deviation. Categorical variables are reported as counts and proportions. Baseline data were defined as the time of ICU admission and included demographic information, and calculation of established risk scores for critically ill patients. The pre-CS therapy period was defined as the 24 h prior to the start of CS therapy and included assessments for vasopressor use, fluid balance, and all laboratory parameters captured as part of routine care. The post-CS therapy period was defined as the 24 h following the end of CS therapy for vasopressor use and fluid balance, the earliest available measurements for lab and blood gas values, and SOFA score. Absolute change from the pre-CS period was calculated as the post-CS measurement minus the pre-CS value. The Wilcoxon signed-rank test was used to compare paired pre- and post-CS data to assess changes associated with CS treatment. P-values less than 0.0001 were reported as <0.0001 in tables and/or figures; all other p-values were rounded and displayed using two decimals. All analyses were performed using SAS 9.4 (SAS, Cary, NC, USA).

## Results

### Patient characteristics

A total of 280 patients from 20 sites in Germany, Spain, Italy and Portugal were enrolled in the Registry up to March 21, 2025. This analysis focuses on the 140 patients who had indication of septic shock for CS therapy. Table 1 summarizes patients’ characteristics including division into ICU survivors and non-survivors.

**Table 1:** Patient characteristics for all patients with septic shock, divided into ICU survivors and ICU non-survivors; Continuous variables are reported as median [IQR], or mean ± standard deviation. Categorical variables are reported as counts and proportions.

Parameter	All Sepsis Patients (n = 140)	ICU Survivors (n = 92)	ICU Non-Survivors (n = 44)
Age (years)	60.5 ± 15.2	58.9 ± 16.2	63.4 ± 13.0
Gender: Male	67.1%	69.6%	61.4%
Gender: Female	32.9%	30.4%	38.6%
Charlson Score	4.0 [2,5]	3.0 [2.0, 5.0]	5.0 [2.0, 7.0]
APACHE II Score	24 [18,30]	23.0 [18.0, 28.0]	25.0 [18.0, 32.0]
SOFA Score (pre-CS)	13 [11,15]	13.0 [11.0, 15.0]	13.0 [11.0, 15.0]

Mortality data available on 136 patients. Legend: ICU: Intensive Care Unit; APACHE: Acute Physiology and Chronic Health Evaluation; SOFA: Sequential Organ Failure Assessment; CS: CytoSorb®.

### Treatment modalities of CytoSorb® therapy in septic shock

Mean number of adsorbers used per patient was 2.8 ± 2.2, with 47% of patients receiving three or more devices. CS was included in the following platforms: kidney replacement therapy (KRT, 85%), standalone hemoperfusion (10%) or extracorporeal membrane oxygenation (ECMO, 5%). Among patients who used KRT as the host circuit for CS, 21.4% of them had ECMO running concomitantly during CS therapy. Among patients who used ECMO as the CS host circuit, 85.7% of them had KRT running concomitantly during CS therapy. Among patients who used either KRT, ECMO or hemoperfusion as the CS host circuit, 2.2% of them also received a plasmapheresis procedure concomitantly with CS therapy. Mean duration of overall CS use was 52.3 ± 53.4 h.

### Biomarkers

Table 2 displays the results of various biomarkers before and after CS therapy. Of note, in patients where CS was integrated into ECMO or used in hemoperfusion mode without KRT, there was also a significant decrease in both lactate from 2.1 [1.2, 2.6] to 1.3 [1.1, 1.4] mmol/L (p = 0.004) and creatinine from 2.1 [1.3, 3.0] to 1.2 [0.7, 1.9] mg/dL (p = 0.0002).

**Table 2:** Overview of different laboratory findings before versus after CS therapy.

Biomarkers	Median levels [IQR] before CS	Median levels [IQR] after CS	p-value
Interleukin 6 pg/mL	2,013 [219, 39,988]	108 [75, 1,662]	<0.0001
C-Reactive Protein mg/dL	15 [3.9, 24.5]	12 [4.2, 18.5]	0.0135
Procalcitonin ng/mL	12.9 [3.8, 68]	4.1 [2.7, 21]	<0.0001
pH	7.36 [7.29, 7.42]	7.4 [7.35, 7.46]	<0.0001
Lactate mmol/L	2.5 [1.6, 5.8]	1.5 [1.1, 2.3]	<0.0001
Creatinine mg/dL	2.0 [1.4, 3.1]	1.3 [0.8, 1.8]	<0.0001

Legend: CS: CytoSorb; IQR; Interquartile range.

### Organ function

**Fig. 1:**
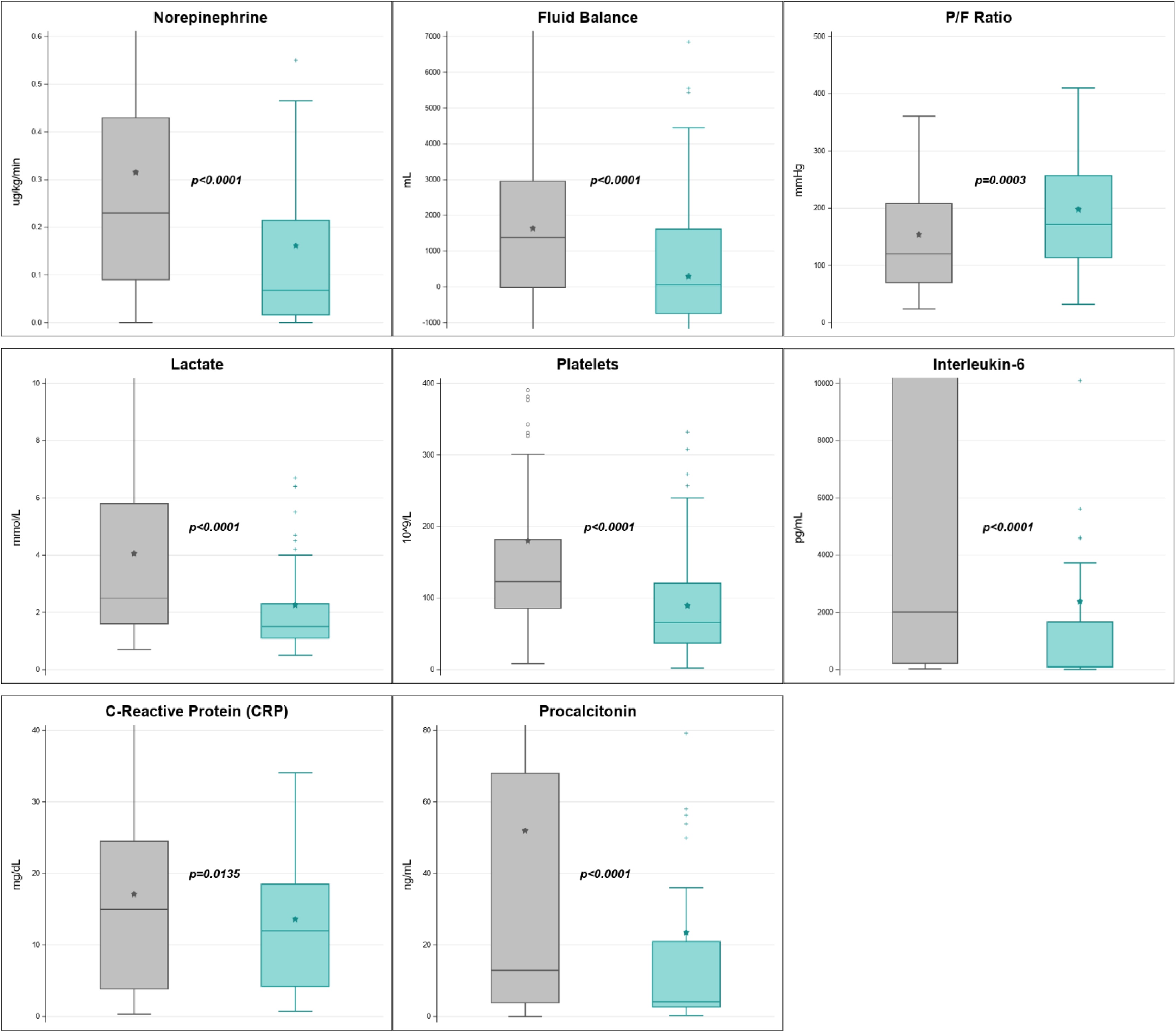
Changes in norepinephrine, fluid balance, P/F ratio, lactate, platelets, interleukin-6, C-Reactive Protein and Procalcitonin in the 24 h period before (grey) versus after CytoSorb® treatment (green) in patients with septic shock. Data are presented as median and interquartile range. For clarity, some graphs were truncated due to extremely high values. Legend: P/F ratio: ratio of partial pressure of oxygen in arterial blood to the fraction of inspiratory oxygen concentration

Median fluid balance improved from +1,386 [−15, 2,960] mL/24 h before treatment to +59 [−738, 1,614] mL/24 h after treatment, while median norepinephrine (NE) requirements decreased from 0.23 [0.09, 0.43] to 0.07 [0.02, 0.22] µg/kg/min (p < 0.0001 for both). Mean arterial pressure (MAP) increased from 73 [67, 81] to 77 [71, 82] mmHg (p = 0.0183), while the MAP/NE index increased from 277 [171, 570] to 795 [303, 1,486] (mmHg × min × kg) / µg (p < 0.0001). Oxygenation, as indicated by P/F ratio, improved significantly from 120 [70, 208] to 172 [114, 257] (p = 0.0003, see Fig. 1). The percentage of invasive mechanical ventilation use was 75.0% pre-CS and 80.3% post-CS therapy, p = 0.489.

Overall SOFA score did not change significantly over the course of CS treatment (p = 0.65). However, there was a significant decrease in sub-SOFA scores for the respiratory and cardiovascular systems as well as for renal function (see Table 3). Coagulation score worsened significantly, secondary to reduced platelet counts.

**Table 3:** Change of sub-SOFA scores over the course of CS treatment.

Sub-SOFA	Parameter for score calculation	N*	Mean score ± SD before CS	Mean score ± SD after CS	Delta Mean	p-value
Respiratory System	P/F ratio	99	3.0 ± 1.0	2.7 1.0	−0.36	0.005
Cardiovascular System	Mean arterial pressure OR administration of vasopressors required	121	3.4 ± 1.0	2.8 ± 1.3	−0.60	<0.0001
Renal Function	Creatinine	112	1.5 ± 1.0	0.8 ± 0.8	−0.73	<0.0001
Coagulation	Platelets	117	1.1 ± 1.1	2.0 ± 1.2	+0.96	<0.0001
Liver	Bilirubin	108	1.2 ± 1.1	1.3 ± 1.1	+0.1	0.42
CNS	GCS	113	2.4 ± 1.8	2.4 ± 1.8	+0.08	0.48

N = number of patients with both pre- and post-CS score available. “Before CS” refers to 24 h period before CS and “after CS” refers to 24 h period after CS. Values are depicted as mean ± standard deviation (SD). Delta Mean = Mean score after CS – Mean score before CS. A negative difference equals an improvement after CS therapy.

Legend: CS: CytoSorb®; SOFA: Sequential organ failure assessment score; CNS: Central nervous system; GCS: Glasgow Coma Scale.

### Mortality

Observed ICU mortality was 32.4%. When the interval between onset of shock (admission to ICU) and start of CS treatment was delayed by at least 1 day, mortality was numerically but not significantly higher compared with immediate start (34 of 100 died (34.0%) vs. 9 of 34 died (26.5%), p = 0.42). For patients who survived, median ICU stay was 23 [14–42] days. In 80 patients with 90-day follow-up data available, 8 (10%) were still requiring kidney replacement therapy (KRT).

### Data quality

Data completeness was 94.2%, as indicated by the automated EDC report (Suppl. Table S1 and Suppl. Fig. S1). Of all queries asked, 97% were answered, closed or "invalid" whereas 3% were still to be answered (see Suppl. Table S2). These 3% open queries are divided among 15% of the patients included. These queries encompass comprehensive data verification and thorough data integrity checks, addressing both consistency and all relevant monitoring processes. Additionally, on-site data monitoring with source data verification has already been conducted at six sites, and this comprehensive process is continuing.

### Safety

No serious device-related adverse effects or device deficiencies were reported in this septic shock cohort, nor have any been reported from the overall COSMOS Registry to date. In terms of laboratory parameters, median albumin levels decreased from 2.6 [2.3, 2.9] to 2.4 [2.0, 2.8] g/dL (p = 0.0116), with 42% of septic shock patients receiving albumin substitution during the course of CS treatment. Median platelet counts decreased from 123 [86, 182] to 66 [37, 121] ×10^9^/L (p < 0.0001).

## Discussion

This interim analysis included 140 patients with septic shock which is the most common indication for CS treatment within the COSMOS Registry. Our analysis found that CS therapy was associated with significant reductions in inflammatory biomarkers and was associated with significant improvements in organ function evidenced in both laboratory parameters such as lactate levels and serum creatinine, and clinical parameters such as decreased norepinephrine requirements and improved fluid balance and oxygenation. The safety profile of the treatment was favorable, with no serious adverse events reported, although platelet counts decreased significantly.

The observed reductions in norepinephrine requirements are consistent with previous studies [3,5] as well as a meta-analysis [8]. Hemodynamic stabilization is a critical factor in managing septic shock, as inadequate perfusion is a cornerstone in the development of multi-organ failure and increased mortality [17]. The decrease in norepinephrine requirements in this study suggests that CS may help restore circulatory function by removing inflammatory mediators that contribute to vasodilation and endothelial dysfunction, both of which are key drivers of hemodynamic instability in septic shock [18]. The reduction in lactate levels may point towards a positive secondary effect of CS therapy indicating improved microcirculation and tissue perfusion, already observed in other studies [19]. However, these findings must be interpreted cautiously as observed associations that, although plausible, are not proof of a treatment effect. The observed reduction in fluid requirements, a major concern in septic shock management, also highlights the potential of CS for reversing endothelial dysfunction and capillary leak and facilitating more restrictive fluid management [20]. The observed improvement in oxygenation reflected by the significantly increased P/F ratio, might be at least partly achieved by fluid restriction and is consistent with results from other trials examining CS in ARDS [21,22]. Despite the observed improvements in hemodynamic stability and lung function, there was no significant change in SOFA score. However, in the sub-SOFA scores, there was significant improvement for lung, cardiovascular and renal function score, while the coagulation score, consisting of platelet counts only, worsened over the course of treatment. Glasgow Coma Scale (GCS) score and liver sub-score did not change significantly although an improvement has been described before [16,23]. This might be explained by the typically short observation window of only a few days, only comprising the duration of CS treatment. Adequately powered randomized clinical studies will be required to determine whether the current observations represent causal treatment effects of CytoSorb® therapy.

The median baseline APACHE II score in this septic shock cohort was 24, corresponding to an estimated mortality rate of 40–50 % for non-surgical ICU patients [24]. Given that APACHE II risk score is considered outdated and may therefore overestimate mortality in contemporary cohorts [25], any comparison of the observed mortality in the current Registry to predicted mortality from standardized risk scores cannot be interpreted as evidence of a treatment effect. Median SOFA score was 13. A 2020 meta-analysis demonstrated that, in patients with septic shock, already a SOFA score of 12 corresponded to an approximate 30-day mortality rate of above 40% [26]. The ICU mortality rate of 32.4% in our Registry analysis is numerically lower but cannot be directly compared with their reported 30-day mortality and in the absence of a control group no conclusions regarding treatment efficacy can be drawn. A propensity-score-weighted study by Brouwer et al., showed improved short- and long-term survival in CS treated patients compared to historical controls [4,27]. Similarly, a retrospective matched cohort study demonstrated a significantly reduced in-hospital mortality and faster weaning from vasopressors in patients undergoing renal replacement therapy with the addition of CS [5]. Furthermore, these findings are supported by a recent systematic review and meta-analysis focused on CS use exclusively in septic shock, which aligns closely with the COSMOS study population in terms of disease severity and treatment context [28]. The meta-analysis, encompassing 744 critically ill patients (449 treated with CS), demonstrated a significant reduction in both in-hospital and 28–30-day mortality. Specifically, CS therapy was associated with a 36% reduction in in-hospital mortality (OR = 0.64 [95% CI: 0.42–0.97], p = 0.036), and a 51% reduction in 28–30-day mortality (OR = 0.46 [95% CI: 0.28–0.78], p = 0.003). These mortality benefits were accompanied by a consistent and significant reduction in vasopressor requirements across the majority of included studies, suggesting meaningful hemodynamic stabilization and shock reversal. In contrast, a previously conducted matched-pair analysis [29] as well as an earlier meta-analysis including studies with a more unselected patient population [30] reported higher mortality in the CS group compared to controls. These contradictory results are also reflective of the complex and heterogeneous nature of sepsis, where therapeutic interventions, including CS, may provide substantial benefit, but additional patient characteristics and treatment timing and dosing also influence outcomes. According to a recent best practice recommendation, CS therapy is most effective when initiated early, preferably within the first 12 h following the diagnosis of septic or vasoplegic shock [14]. Furthermore, as recently emphasized by the study of Berlot et al., the effectiveness of CS appears to be clearly influenced by early initiation and appropriate dosing, underscoring the importance of optimizing timing and treatment intensity in clinical practice [31]. This is in line with our subgroup analysis that revealed a signal of better outcome if CS was started early after onset of shock, although this did not reach statistical significance in our population. The importance of early CS start is further supported by findings that lactate levels greater than 6 mmol/L, may serve as potential predictors of worse outcomes, underscoring the importance of timely intervention before irreversible organ damage occurs [4,5,27].

Although no SADE or DD were reported in the study cohort of the Registry, the decrease in platelet count during the course of CS therapy in this study is noteworthy. It is important to recognize that thrombocytopenia is a well-documented complication of sepsis, often resulting from increased platelet consumption due to widespread inflammation, endothelial dysfunction, and the formation of microthrombi [33,34]. Additionally, sepsis-induced disseminated intravascular coagulation (DIC) can lead to platelet activation and depletion [35]. Furthermore, in a recent multicenter retrospective cohort of 1,413 adults with acute kidney injury on CKRT, platelet counts declined by about 14% during CKRT , indicating that thrombocytopenia can occur as an effect of CKRT itself [36]. The CYTOSOLVE study in 29 critically ill patients also found significant reductions in platelet levels with CS and CKRT. However, the lack of pre/post-adsorber measurements prevented isolating CS’s direct effect from other factors such as underlying disease and the use of CKRT [37]. Despite statistical significance, the potential clinical relevance remains unclear, as no adverse events such as bleeding were reported [38]. In a recent real-world analysis of CS use in cardiogenic shock, a significant platelet decline was observed during therapy from a median of 140 × 10^9^/L before to 54 × 10^9^/L after treatment (p < 0.001) [39]. The authors note, however, that this finding should be interpreted cautiously, as many patients also had mechanical circulatory support such as Impella, VA-ECMO and CKRT, all of which have known risks for thrombocytopenia due to mechanical platelet destruction and/or consumption. The same was stated by other authors who could not directly link the CS device to thrombocytopenia [32,40]. Therefore, while platelet counts were reduced in our study after CS use, it is difficult to definitively conclude that it was caused solely from CS since the etiology was likely multifactorial. Regardless, occurrence of thrombocytopenia should be considered as a potential risk with CS use.

Similarly, an additional loss of albumin during CS use cannot be definitively excluded. Although no clinically relevant decrease was observed in our current results, more than 40% of patients received albumin supplementation likely to correct reduced levels resulting from the aggressive fluid resuscitation and resultant hemodilution plus the effects of the underlying critical illness and extracorporeal circulation. Therefore, in the absence of pre- and post-adsorber albumin measurements, the impact of the adsorber on albumin is also not easily discernable. Published evidence regarding albumin reduction during CS therapy are also mixed, as some studies report measurable losses [38], whereas others found no indication of a systematic problem [14].

Given the many influencing factors, such as the underlying critical illness and exposure to other extracorporeal surfaces like CKRT, any observed platelet or albumin decline in studies with CS should be interpreted with caution, yet it remains an important safety aspect that warrants close monitoring.

### Clinical implications

By effectively removing overshooting inflammatory mediators such as cytokines and DAMPs, CS may help restore endothelial integrity and reduce capillary leak – key pathophysiologic contributors to fluid overload and hemodynamic instability in septic shock [18]. As a result, patients may experience both a reduction in positive fluid balance and decreased vasopressor requirements. Importantly, the co-occurrence of these two benefits in this study suggests that improved fluid management was not achieved at the expense of hemodynamic support. The observed hemodynamic stabilization, coupled with lower cumulative fluid balance during treatment, underscores the potential of CS to support the critical goal of early fluid de-resuscitation in septic shock. Clinical outcomes are likely influenced by timely initiation of therapy, with prior studies demonstrating less favorable results when treatment is delayed [23,41,42]. While this analysis did not yet evaluate CS dosing strategies, emerging evidence suggests that the number of adsorbers and treatment intervals may also impact outcomes [31,43].

### Limitations

The most notable limitation applicable to all observational intervention registries such as COSMOS is the lack of a control arm, making it impossible to determine definite causality between the intervention and the observed outcomes and therefore the results should be viewed only as plausible associations. This limitation is particularly relevant when interpreting the observed biomarker and hemodynamic improvements that may simply represent the natural course of recovery rather than a direct CS treatment effect. An additional limitation is that in some of the subjects included in this analysis, some datasets were incomplete at the time of data extraction thereby resulting in a smaller sample size for certain analyses.

## Conclusions

Real-world outcomes with the use of CS as part of standard clinical care reported in the current analysis indicate significant improvements in key therapeutic outcomes in the management of septic shock. Specifically, CS therapy was associated with significant reductions in norepinephrine and fluid requirements as well as improvements in oxygenation. Device use was generally safe, although a significant drop in platelet counts was observed. The observational nature of the Registry requires caution when interpreting the results and randomized controlled trials will be necessary to confirm the treatment effects observed.

## CRediT authorship contribution statement

Conceptualization: Fabio Silvio Taccone, Teresa Klaus, Joerg Scheier, Thomas Kirschning and Ricard Ferrer; Methodology: Fabio Silvio Taccone, Teresa Klaus, Thomas Kirschning and Ricard Ferrer; Writing – original draft preparation: Teresa Klaus, Thomas Kirschning and Ricard Ferrer; Writing – review and editing: Teresa Klaus, Thomas Kirschning, Moritz Unglaube, Ulf Guenther, Julian Kreutz, Matthias Thielmann, Andreas Baumann, Andreas Kribben, Dietrich Henzler, Nuno Germano, Aschraf El-Essawi, Filippo Aucella, Thomas Guenther, Martin Bellgardt, Bartosz Tyczynski, P. Christian Schulze, Dana Tomescu, Gabriella Bottari; Statistics: Weihong Fan; Visualization: Teresa Klaus, Weihong Fan; Supervision: Fabio Silvio Taccone, Jorge Hidalgo, Jean-Louis Teboul, Joerg Scheier, Efthymios N. Deliargyris, Ricard Ferrer. All authors have read and agreed to the published version of the manuscript.

## Informed consent

Informed consent was obtained from all subjects involved in the study.

## Ethics approval and consent to participate

The Registry is being conducted in accordance with ISO 14155:2020, the Declaration of Helsinki, and Good Clinical Practice guidelines as outlined in the International Conference on Harmonization E6, along with all applicable local regulations. Prior to initiation, the study protocol, informed consent form (ICF), and any recruitment-related documents, including advertisements, must receive approval from the Institutional Review Board (IRB) or Independent Ethics Committee (IEC). Any protocol amendments or changes to the ICF will also require IRB/IEC approval. The initial ethics approval number for University Hospital Vall D'Hebron in Barcelona, Spain is PR(AG)86/2022. Each participating center must obtain approval from its local IRB before the study can be initiated.

## Funding

CytoSorbents Corporation and CytoSorbents Medical Inc. are the funding sources.

## Availability of data and material

The data are available upon reasonable written request and with written permission of the CytoSorbents Corporation. Data Management Plan and Informed Consent Forms are available upon request.

## Declaration of competing interest

Fabio Silvio Taccone, Gabriella Bottari, Jorge Hidalgo, Andreas Kribben, Jean-Louis Teboul, Dana Tomescu and Ricard Ferrer have consulting contracts with CytoSorbents Corporation and CytoSorbents Medical Inc. Andreas Baumann received reimbursements for travelling expenses and honoraria for presentation from CytoSorbents Europe GmbH. Julian Kreutz received honoraria for presentation from CytoSorbents Europe GmbH. Teresa Klaus and Joerg Scheier are full-time employees of CytoSorbents Europe GmbH. Efthymios N. Deliargyris and Weihong Fan are full-time employees of CytoSorbents Corporation and CytoSorbents Medical Inc.
