# Supplementary material for: The International, Prospective COSMOS (CytOSorb® TreatMent Of Critically Ill PatientS) Registry: Interim Results in Patients with Septic Shock
Source: Ann Intensive Care. 2026 Mar 19;16:100052. doi: 10.1016/j.aicoj.2026.100052 (PMC13015761; doi:10.1016/j.aicoj.2026.100052)
Supplement: Supplementary file 1 [file mmc1.docx]

**Supplemental Material**


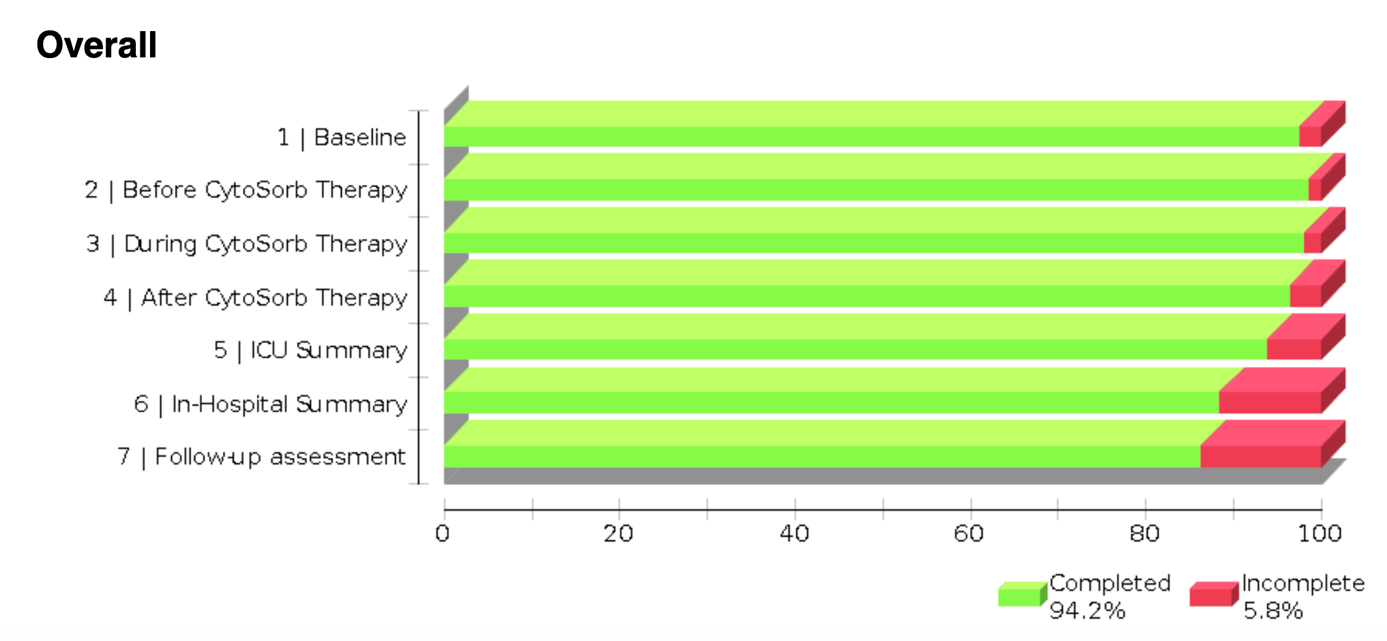


Suppl. Figure 1. Overview of data completeness from built-in EDC report


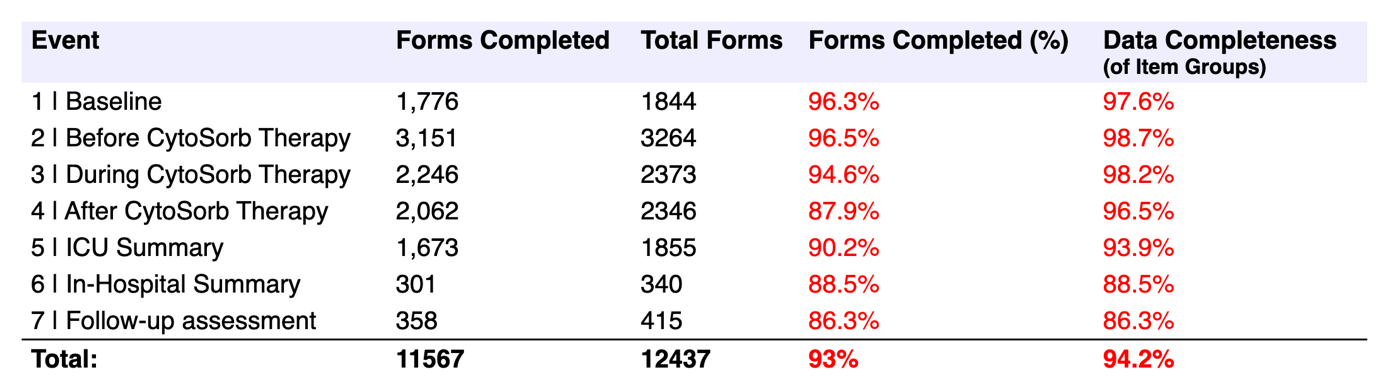


Suppl. Table 1. Overview of data completeness from built-in EDC report

| **Ratio** | | **Absolute numbers** |
| --- | --- | --- |
| Answered Queries / all Queries | 97,4% | 9217/ 9463 |
| Open Queries / all Queries | 2,6% | 246/ 9463 |
| Subjects with Queries / all Subjects | 15,4% | 59/ 383 |

Suppl. Table 2. Number and ratio of solved queries in registry
